# Genetic changes observed in a case of adult pilocytic astrocytoma revealed by array CGH analysis

**DOI:** 10.1186/s13039-014-0095-2

**Published:** 2014-12-23

**Authors:** Nives Pećina-Šlaus, Kristina Gotovac, Anja Kafka, Davor Tomas, Fran Borovečki

**Affiliations:** Laboratory of Neurooncology, Croatian Institute for Brain Research, School of Medicine University of Zagreb, Šalata 12, 10000 Zagreb, Croatia; Department of Biology, School of Medicine, University of Zagreb, Šalata 3, 10000 Zagreb, Croatia; Department for Functional Genomics, Center for Translational and Clinical Research, University of Zagreb School of Medicine, and University Hospital Center Zagreb, Šalata 2, 10 000 Zagreb, Croatia; Department of Pathology, School of Medicine, University of Zagreb, Šalata 10, 10000 Zagreb, Croatia; Hospital Center “Sisters of Charity”, Vinogradska 29, 10000 Zagreb, Croatia; Department of Neurology, University Hospital Center Zagreb, Kišpatićeva 12, 10000 Zagreb, Croatia

**Keywords:** Pilocytic astrocytoma, aCGH analysis, Copy number aberrations

## Abstract

**Background:**

A palette of copy number changes in a case of adult pilocytic astrocytoma analyzed by Array Comparative Genomic Hybridization (aCGH) is presented. Pilocytic astrocytomas are specific gliomas that are benign and biologically distinct and the molecular mechanisms responsible for their development remain unexplained. The aCGH was performed using SurePrint G3 Human CGH microarrays 4 × 180 K (Agilent Technologies). To ascertain whether some of the aberrations were of constitutive nature, we also analyzed the blood sample from the same patient.

**Results:**

The result of aCGH analysis demonstrated differences in the tumor tissue when compared to normal control on the array and also to autologous DNA from patient’s blood. The total number of aberrations found in our case was 41 including 37 deletions and 4 amplifications. Whole chromosomal gains and losses were not observed. Collectively, our results showed three deletions and one amplification at 1p, two deletions at 2q, two deletions at 4q, two deletion at 5q, two deletions at 7p and two deletions at 7q; there were also three deletions at 8q, one deletion at 9p, one deletion at 10p, three deletions and one amplification at 10q. Chromosome 11 showed two deletions at 11p, while there was one deletion at 12p and one at 12q. Four deletions at 14q; two deletions at 15q, one amplification at 17q and one deletion at 17q; one deletion at 18p, two deletions at 22q and finally one deletion at Xp and one deletion and one amplification at Xq. Among the signaling pathways, olfactory transduction, Fc gamma R-mediated phagocytosis and p53 signaling pathway showed significant enrichment ascertained by gene ontology (GO) analysis using the DAVID software.

**Conclusions:**

Our aCGH analysis is bringing subtle genomic alterations thus broadening genetic spectrum of adult pilocytic astrocytoma in order to offer new molecular biomarkers that will help in diagnostics and therapeutic decision-making.

## Background

Astrocytic brain tumors are the most common primary central nervous system neoplasms, but despite recent advances in glioma genetics [1-3], the molecular mechanisms behind their development and progression remain largely unexplained. Different subtypes of gliomas differ significantly in their age distribution, growth potential, tendency for progression and clinical course. Astrocytomas are classified according to their lineage of origin, histology, behavior and prognosis into four WHO grades [4,5]. Pilocytic astrocytomas (PA) most commonly develops during the first two decades of life, but may also develop in the third or fourth decade, with relatively few arising in patients older than 50 years [6,7]. PA are clinically, biologically, and histologically distinct from WHO grade II-IV gliomas. PA typically shows benign clinical behavior and malignant progression is extraordinarily rare, describing PA as a benign tumor. Adult pilocytic astrocytomas (PAs) are rare and usually have an aggressive clinical course compared with pediatric patients [8,9]. Due to the heterogeneity of histological features and lack of tumor specific immunohistochemical, cytogenetic and molecular markers, great effort has been put into molecular and translational research of these tumors in the last few years [10-14]. Another point for the necessity of molecular characterization is the fact that although the PAs can be cured with complete surgical resection, clinical evidence demonstrate that unfavorable anatomical location may impair complete resection and lead to progression or recurrence.

Array comparative genomic hybridization (aCGH) is a modern technique for detecting gene copy number variation across the entire genome. It is a reliable and sensitive method and has been widely used in genetic profiling of different types of cancer as one of the main characteristics of cancer diseases is genomic instability [15,16]. We were interested to characterize unbalanced genomic changes in our pilocytic astrocytoma patient and offer potential candidate genes detected by aCGH analysis. Until recently little was known about genetic events that are involved in PA initiation and early development. Few constant genetic findings reported are: the association of neurofibromatosis type 1 (NF1) syndrome with an increased incidence of low-grade gliomas [1], mutations of *KRAS* activating MAPK pathway in sporadic PA [17], mutations of *BRAF*, especially a substitution called V600E, and constant chromosome gains at 7q34 [18-22].

Here we present aCGH analysis of a case of adult pilocytic astrocytoma.

### Patient and methods

Pilocytic astrocytoma brain tumor was collected from the Department of Neurosurgery, University Hospital “Sisters of Charity”, Zagreb, Croatia. Autologous blood sample of the same patient was also obtained. The patient had no family history of brain tumors. The tumor tissue was frozen in liquid nitrogen and transported to the laboratory, where it was immediately transferred to −75°C. The peripheral blood sample was collected in EDTA and processed immediately. Magnetic resonance imaging (MRI) revealed that the astrocytic brain tumor was localized in right frontoparietal region. During the operative procedure the tumor was removed using a microneurosurgical technique. On the basis of the pathohistological findings diagnosis of pilocytic astrocytoma was established.

Ethical approval was received from the Ethical Committees Medical School University of Zagreb and University Hospital “Sisters of Charity”, and the patient gave his informed consent.

### DNA extraction

Approximately 0.5 g of tumor tissue was homogenized with 1 ml extraction buffer (10 mM Tris HCl, pH 8.0; 0.1 M EDTA, pH 8.0; 0.5% sodium dodecyl sulfate) and incubated with proteinase K (100 μg/ml; Sigma, USA; overnight at 37°C). Phenol chloroform extraction and ethanol precipitation followed. Blood was used to extract leukocyte DNA. Five ml of blood was lysed with 7 ml distilled water and centrifuged (15 min/5000 *g*). The pellet was then processed as for DNA extraction from the tissue samples. Samples were purified using PCR purification kit (Qiagen). The concentrations were measured by Nanodrop and the purity of DNA was determined. Each DNA sample was analyzed on 1.5% agarose gel to assess genomic DNA intactness and the average molecular weight.

### aCGH

Array Comparative Genomic Hybridization (aCGH) was performed using SurePrint G3 Human CGH microarrays 4 × 180 K (Agilent Technologies) following the manufacturer’s instructions. Briefly, 1 μg of genomic DNAs corresponding to either a human reference control (Promega) or test samples were fragmented by heating at 95°C for 10 minutes. Fragmented DNAs were labeled with Cy3 (reference DNA) and Cy5 (test samples) fluorescent dUTP, respectively, using the SureTag Complete Labeling Kit (Agilent Technologies). Purification columns (Agilent) were used to remove the unincorporated nucleotides and dyes. The labeled samples along with human Cot-1 DNA were added together and hybridized on the array slides. Hybridizations of labeled DNAs to SurePrint G3 Human CGH Arrays (4 × 180 K) (Agilent Technologies) were performed in a hybridization oven at 65°C at 20 rpm for 24 hours. The slide was scanned at 3 μm resolution on Agilent Microarray Scanner System (Agilent Technologies). Agilent CytoGenomics software (Agilent Technologies) was used to visualize, detect, and analyze chromosomal patterns within the microarray profiles.

## Case presentation

**Figure 1:**
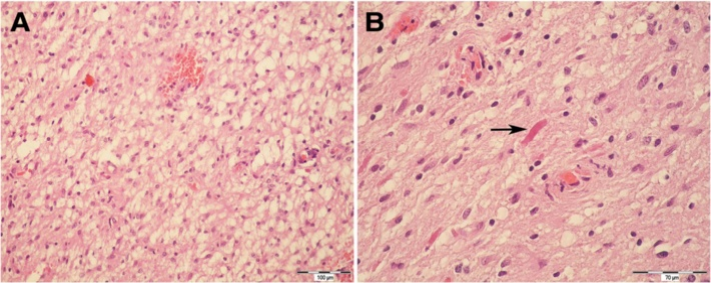
**The tumor areas with microcystic-clear cell features were prominent (A, 200 × HE).** Rosenthal fibers (arrow) were focally present, predominantly in loosely arranged areas of tumor (**B**, 400 × HE).

Our patient was a 42 years old male admitted to the Department of Neurosurgery, University Hospital “Sestre Milosrdnice” manifesting symptoms of raised intracranial pressure including severe occipital headaches and nausea caused by a brain tumor. The symptoms lasted for 10 months. The patient was without clinical NF1 or NF2 and had no family history of brain tumors. Using the magnetic resonance imaging (MRI) a tumor lesion was found in the right anterior intraventricular region. The well circumscribed tumor was located from the III ventricle all the way to the foramen of Monro and cranially to the corpus callosum. During the operative procedure the astrocytoma was maximally reduced using a microneurosurgical technique. The surgical access route was the interhemispheric transcallosal approach, passing through the corpus callosum and entering intraventricularly in the corpus of the right lateral ventricle. The foramen of Monro was freed to the right and the communication between right lateral ventricle and the third ventricle was established. On the frozen and subsequent permanent sections tumor was made of alternating areas of densely and loosely packed fibrillary neoplastic cells with long, hair-like cytoplasmic processes. Microcystic-clear cell features (Figure 1A) and Rosenthal fibers (Figure 1B) were also present. In some areas hyalinized blood vessels and glomeruloid microvascular proliferation was also observed. Mitoses, anaplastic cells as well as necrosis were not found. Immunohistochemically, the tumor cells were strongly positive for glial fibrillary acidic protein (GFAP). Neuropathological examination revealed pilocytic astrocytoma and classified the tumor into grade I according to the WHO criteria [23]. The follow-up was 10 years without recurrence.

**Figure 2:**
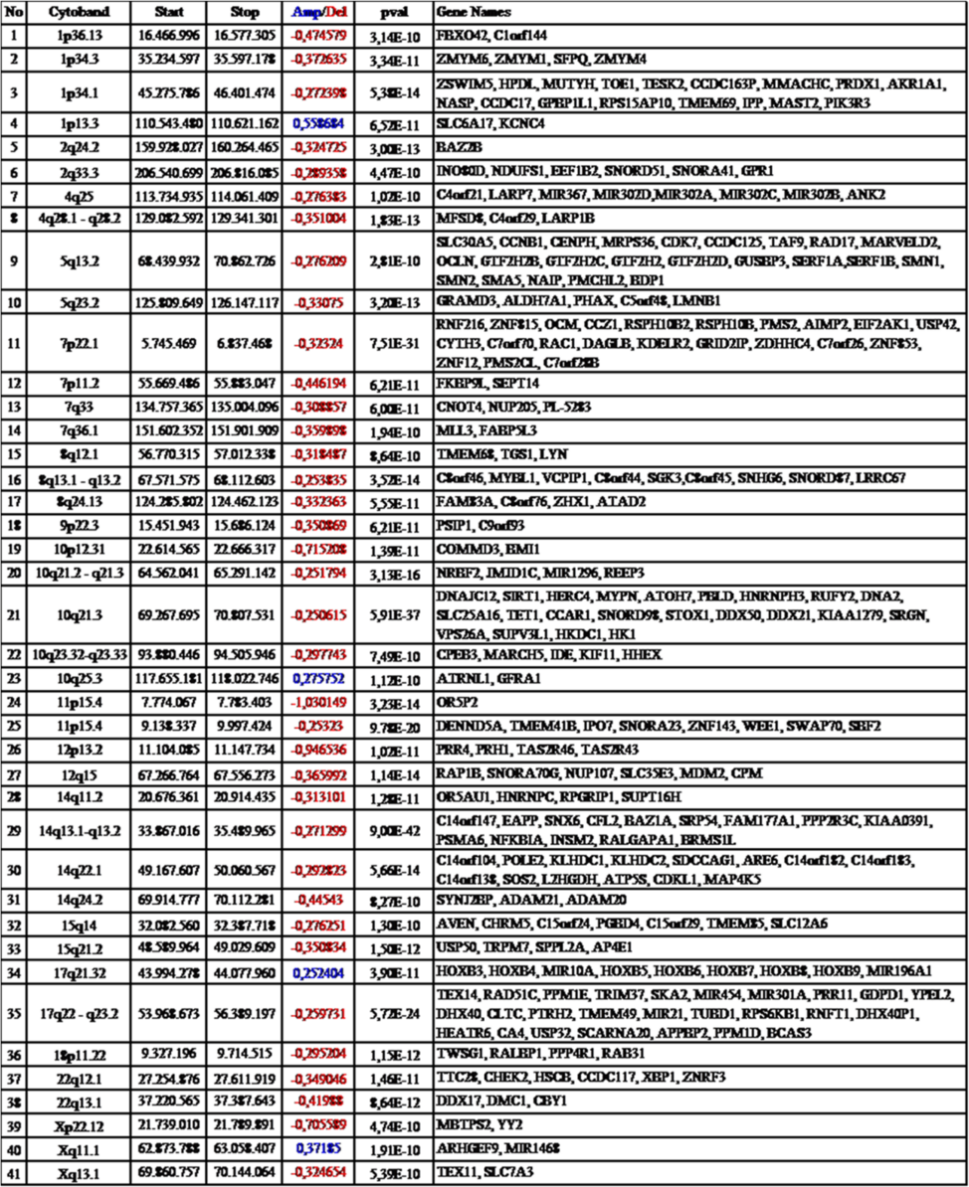
**The major genomic findings and annotated genes in adult pilocytic astrocytoma.**

The result of aCGH analysis demonstrated differences in the tumor tissue when compared to normal control on the array. The total number of aberrations found in our case was 56, among which deletions dominated. Whole chromosomal gains and losses were not observed. To ascertain whether some of the aberrations were of constitutive nature, we also analyzed the blood sample from the same patient compared to the reference DNA. DNA obtained from blood comprised altogether 22 copy number changes of which there were 13 deletions and 9 amplifications. The majority of changes (68%) from autologous constitutive DNA were repeated in the belonging tumor DNA (15/22). Four amplifications and 3 deletions were exclusive for the blood DNA. The analyzed DNA from pilocytic astrocytoma contained altogether 41 copy number alterations exclusively found in tumor, including 37 deletions and 4 amplifications. The observed major genomic findings are detailed in Figure 2.

Collectively, our results showed three deletions and one amplification at 1p, two deletions at 2q, two deletions at 4q, two deletions at 5q, two deletions at 7p and two deletions at 7q; there were also three deletions at 8q, one deletion at 9p, one deletion at 10p, three deletions and one amplification at 10q. Chromosome 11 showed two deletions at 11p, while there was one deletion at 12p and one at 12q. Four deletions at 14q; two deletions at 15q, one amplification at 17q and one deletion at 17q; one deletion at 18p, two deletions at 22q and finally one deletion at Xp and one deletion and one amplification at Xq.

Annotated genes possibly involved in this tumor are also presented in Figure 2. Pseudogenes and loci with unknown gene function were excluded from the table.

**Figure 3:**
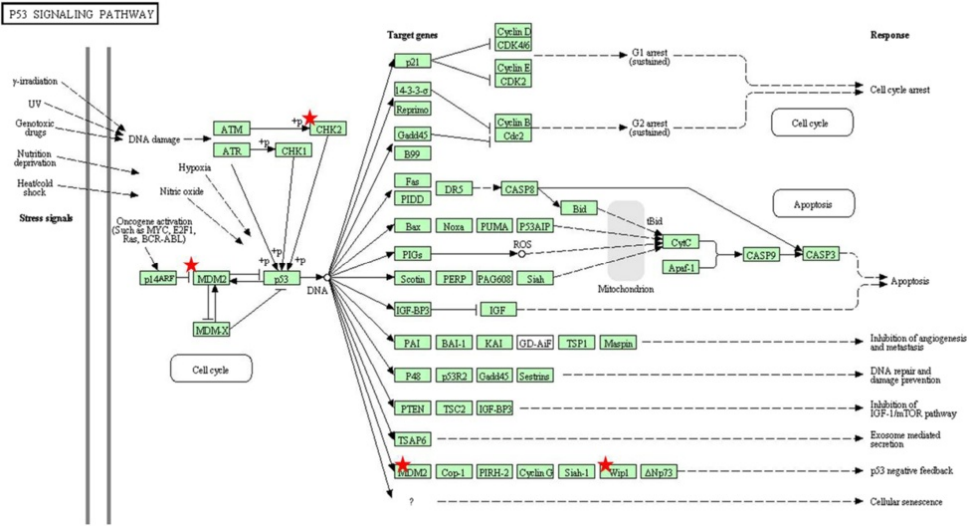
**Gene ontology (GO) analysis using the DAVID software.** Role of the affected genes within the p53 signaling pathway.

In an effort to ascertain possible overrepresentation of specific gene ontology (GO) terms among the genes located within the regions showing altered DNA copy numbers, we performed GO analysis using the DAVID software. The GO terms related to the genes located within the regions with altered copy number included olfactory and sensory transduction, cognition, zinc finger region, neurological system process, G-protein coupled receptor, DNA repair and proto-oncogene. Among the signaling pathways, olfactory transduction, Fc gamma R-mediated phagocytosis and p53 signaling pathway showed significant enrichment (Figure 3).

To summarize the functions of candidate annotated genes we found changes in chromosomal regions harboring genes whose products are transcription factors, activators or repressors of transcription, translation factors, oncogenes, those involved in DNA repair, in cytoskeletal organization, development, chromatin remodeling, apoptosis. There were also several microRNAs, kinases, neurotransmitters, tumor suppressor genes and methyltransferases. Since the etiology of pilocytic astrocytoma (PA) is associated to several major cellular signaling pathways we investigated whether some of the annotated genes belong to pathways specific to PA. We found genes that are involved in Ras-Raf-MEK-ERK signaling, p53 signaling, RB signaling, Wnt signaling.

## Discussion

Although the PAs are well circumscribed, benign and show prolonged overall survival and high complete remission rates, some are surgically inaccessible, leading to chronic disease with recurrences. As technologies progress genetic profiles and molecular findings have become recognized as potential markers of clinical distinction of tumor subtypes. Molecular characteristics are also being helpful in explaining the responses to therapy [5].

In order to investigate genetic alterations in a patient with pilocytic astrocytoma we performed aCGH analysis using DNA samples obtained from patient blood and tumor. Array CGH compares genomic DNAs isolated from test and reference samples that are differentially labeled with red (Cy5) and green (Cy3) fluorescent dyes and competitively hybridized to known mapped segments of human genomic DNA (eg, bacterial artificial chromosomes/P1-derived artificial [BAC/PAC] or oligonucleotide probes) attached to a slide. There are several major advantages to CGH arrays, including ease of implementation, the ability to use archival samples, and the ability to detect copy number changes at a higher resolution.

In our patient whole chromosomal gains and losses were not observed which is in accordance with previous findings [24], although some reports on whole chromosomal gains (chromosomes 5,7,8 [25-27]) and losses (chromosomes 7, 8, 17 [5,28]) in PAs have been published. The reported cytogenetic band abnormalities include gains of 1p, 2p, 4q-9q and 13q and losses on 1p, 9q, 12q and 19–22 [5,25,26,29-32]. There are also reports on subtelomeric gains at 7qter, 12qter, 13cen, 19pter, X/Yqter and duplication at 3pter, while subtelomeric deletions have been observed at 8pter, 20pter and 21qter. It is interesting to note that most PAs exhibit a normal karyotype [5,28,33,34]. Nevertheless there are approximately 32% that show chromosomal abnormalities. Moreover, the higher number of chromosomal abnormalities in PAs are associated with older patients, while patients younger than 15 years usually show single chromosomal abnormalities [5,32]. The situation found in our case of adult PA is confirming this since we observed many copy number changes.

Our results also showed that some changes were similar to the reports, but some were unique for our patient.

The high number of copy number changes found in our patient can also be indicative of the acquisition of genomic instability of the tumor, especially since deleted regions harbor genes involved in mismatch repair and therefore the acquisition of genomic instability. Characteristic behavior of PA indicates that they may be molecularly distinct glial neoplasms.

As far as annotated genes possibly involved in this tumor are in question our results bring many candidates. The roles of many of them are still unknown. Identifying narrow regions with altered DNA copy number is an important finding in tumor genetics, as genes mapped in these regions may represent potential candidate tumor suppressor genes and oncogenes. The cytoband 17q11.2 holding the *NF1* gene was not changed in our case which is consistent to nowadays standpoint that *NF1* abnormalities are unique to NF1 associated PAs and are not observed in sporadic tumors indicating that sporadic PAs and the ones associated to neurofibromatosis 1 are molecularly distinct. The abnormalities involving molecular components of Ras superpathway are believed to play a role in sporadic PAs [35,36]. The most common genomic abnormalities found in sporadic PAs are *BRAF* fusion genes. Changes involving *BRAF* seem to be very frequent in sporadic PAs [20,22,37]. There are several reported fusions, of which *BRAF*^*ex9*^*–KIAA1549*^*ex16*^ is the most common, followed by *BRAF*^*ex9*^*–KIAA1549*^*ex15*^ and *BRAF*^*ex11*^*–KIAA1549*^*ex16*^ [38]. Another *BRAF* fusion is the one with *FAM131B* gene [21]. Our sample showed 4 changes on chromosome 7, but the closest cytobands were q33 and q36.1 leaving us to conclude that *BRAF* changes were not involved in our PA case. This is not an unusual finding since [9] report on only 20% of sporadic PA cases with *BRAF–KIAA1549* fusion. There is still the unanswered question on *BRAF* mutational activation in our case.

Of interest is also that many of the annotated genes have been found to be expressed in the human glioma cell line GaMg [39].

Because pilocytic astrocytomas are clinically, biologically, and histologically distinct from WHO grade II-IV gliomas [27], it is interesting to provide information on genetic blueprint of those tumors. Common genetic changes and tumor associated mutations found in higher grade gliomas, *p53*, *PDGF*, *p16* (*CDKN2A*), *IDH1* and *IDH2* are rarely reported in PAs [1] which is consistent to our results that also indicate lack of abnormalities in loci where those genes reside. It is interesting to note that genes *SOCS3*, *HLA-DRalpha*, *HLA-DRB1*, *A2M*, *HIPK2*, *MATN2*, *TIMP1*, *TIMP4*, *tenascin-R*, *FGFR1*, *NTRK2, ErbB4*, *VEGF* and *VEGFR* reported by other authors [2,5,24] to be involved in PA were not found in our patient.

Nevertheless, it is important to validate (verify) the involvement of candidate genes in further studies that employ other methods of molecular biology.

To include intrinsic control is very important due to the possibility of large scale copy number polymorphism in the human genome [40]. Our findings demonstrated that up to 68% of changes observed both in the constitutive DNA were also present in the tumor tissue as compared to unrelated normal reference. This could be explained by copy number polymorphism specific for the person, but it could also be an indication on germline genetic predisposition.

Recently it was shown that age and location also matter in sporadic PA [4,35,41]. Recent data suggest that pediatric and young adult gliomas arise from neurodevelopmental defects that occur during specific developmental windows. BRAF signaling has been shown to be mandatory in the development of the cerebellum, whereas NF1, or more upstream FGFR signaling, seems important for diencephalic and midline development [4]. Interestingly, we found copy number changes in regions that harbor developmental genes i.e. *HOX* genes, *oncomodulin*, *DAGLB*, *ZNF12*, *TRIM 37*, *MBTPS2*, *YY2*.

## Conclusions

Despite many recent advances on the molecular biology of pilocytic astrocytoma, its molecular blueprint of development and progression is still largely unexplained. Our findings contribute to better understanding of human PA brain tumor genetic profile and suggest that copy number alterations play important roles in PA etiology. The findings may provide molecular biomarkers that will help in diagnostics and therapeutic decision-making.

## Consent

Written informed consent was obtained from the patient for publication of this report. A copy of the written consent is available for review by the Editor-in-Chief of this journal.
